# Biological and clinical significance of cancer stem cell plasticity

**DOI:** 10.1186/s40169-014-0032-3

**Published:** 2014-10-07

**Authors:** Yongyou Zhu, Ming Luo, Michael Brooks, Shawn G Clouthier, Max S Wicha

**Affiliations:** University of Michigan Comprehensive Cancer Center, 1500 E. Medical Center Dr., Ann Arbor, 48109 MI USA

**Keywords:** Cancer stem cells, MET, EMT, Plasticity

## Abstract

In the past decade, the traditional view of cancers as a homogeneous collection of malignant cells is being replaced by a model of ever increasing complexity suggesting that cancers are complex tissues composed of multiple cell types. This complex model of tumorigenesis has been well supported by a growing body of evidence indicating that most cancers including those derived from blood and solid tissues display a hierarchical organization of tumor cells with phenotypic and functional heterogeneity and at the apex of this hierarchy are cells capable of self-renewal. These “tumor imitating cells” or “cancer stem cells” drive tumorigenesis and contribute to metastasis, treatment resistance and tumor relapse. Although tumor stem cells themselves may display both genetic and phenotypic heterogeneity, recent studies have demonstrated that cancer stem cells maintain plasticity to transition between mesenchymal-like (EMT) and epithelial-like (MET) states, which may be regulated by the tumor microenvironment. These stem cell state transitions may play a fundamental role in tumor progression and treatment resistance. In this review, we discuss the emerging knowledge regarding the plasticity of cancer stem cells with an emphasis on the signaling pathways and noncoding RNAs including microRNAs (miRNA) and long non-coding RNAs (lncRNAs) in regulation of this plasticity during tumor growth and metastasis. Lastly, we point out the importance of targeting both the EMT and MET states of CSCs in order to eliminate these lethal seeds of cancers.

**Electronic supplementary material:**

The online version of this article (doi:10.1186/s40169-014-0032-3) contains supplementary material, which is available to authorized users.

## Introduction

Despite significant advances over the last decade in the diagnosis and treatment of cancer, the fact remains that, once metastatic, the disease remains almost universally incurable. There is now substantial evidence that many cancers, including breast cancer, are hierarchically organized and driven by a population of cells that display stem cell properties [1]-[3]. These cells have been referred to as tumor-initiating cells (TICs) or cancer stem cells (CSCs). Subsequent studies have provided further evidence that CSCs mediate tumor metastasis and are associated with therapeutic resistance [4]-[7]. On this basis, targeting CSCs holds great potential for limiting tumor growth and metastasis as well as preventing therapeutic resistance and cancer relapse.

The high degree of phenotypic and functional plasticity has been increasingly recognized as one of the significant properties of CSCs driving therapeutic resistance. We recently reported that breast CSCs display two reversible phenotypic and functional states: an EMT, epithelial-to-mesenchymal transition state and an MET, mesenchymal-to-epithelial transition state [8]. The transition between these two states in breast CSCs may facilitate breast cancer growth, dissemination and formation of distal metastasis. In this review, we will discuss emerging aspects regarding the plasticity of CSCs, the signaling pathways that regulate this plasticity during tumor growth and the role of CSC plasticity in tumor progression and metastasis. We will also discuss the clinical significance of targeting CSC plasticity.

## Review

### Cancer stem cells and plasticity

#### The cancer stem hypothesis

The cancer stem hypothesis posits that the majority of human cancers are initiated and maintained by a small population of cells that display stem cell properties including unlimited self-renewal as well as the ability to recapitulate the cellular distribution of the original tumor [9],[10]. These cells were first described in human acute myeloid leukemia [11]. The first evidence for CSCs in solid tumors was presented in 2003 by Al-Hajj et al. who prospectively enriched a population of breast cancer cells with high tumorigenic properties [12]. These breast CSCs were characterized by virtue of their expression profile of specific cell surface markers, including EpCAM^+^, CD24^-^ and CD44^+^. As few as 100 cells bearing this phenotype were capable of forming tumors in immune deficient NOD/SCID mice, whereas 10,000 cells without this cell surface phenotype were non-tumorigenic. An *in vitro* cell culture assay under non-adherent conditions for quantitating the stem/progenitor cell proportion in human mammary epithelial cells has also been described [13]. In this assay, only the cells with stemness are able to proliferate and generate mammosphere structures. More recently, it has been demonstrated that cells high in aldehyde dehydrogenase (ALDH) activity are enriched in breast CSCs, as determined by using the Aldefluor assay(Stem Cell Technologies) [14]. The cancer stem hypothesis and the prospective isolation and characterization of cancer stem-like populations from leukemia, breast cancer and a wide variety of other solid malignancies including that of the brain [15], prostate [16],[17], colon [18],[19], pancreas [20], liver [21],[22], lung [23], and head and neck [24] in the past decade has been one of the major advances in current cancer research. Increasing studies have shown that CSCs display treatment resistance to chemotherapy and radiation therapy [4]-[6],[25], while clinical neoadjuvant chemotherapy expanded the proportion of CSCs [20],[26].

### Epithelial-mesenchymal plasticity of CSCs

In the case of breast cancer, CSC populations identified by the markers CD24^-^CD44^+^ or ALDH^+^ were characterized as minimally overlapping, largely separate cell populations, each capable of initiating tumors in immune deficient mice [14]. However, whether these different phenotypic populations identify distinct or independent CSCs in the tumor remained to be resolved. To further characterize these distinct breast CSC populations, we prospectively isolated these distinct subsets of breast cancer cells from a total of 30 human breast cancer samples. These tumor samples were digested in collagenase to obtain single tumor cells. Following tumor cell disassociation, tumor cell samples were incubated with anti-CD44, anti-CD24, and anti-lineage mixture antibodies (PE-conjugated anti-CD2, CD3, CD10, CD16, CD18, CD31, and CD 140b), and then labeled by Aldefluor assay, and analyzed using MoFlo Astrios flow cytometry. Side and forward scatter were used to eliminate debris and cell doublets, and the Lin^-^ tumor cells were further analyzed and sorted for ALDH^+^, ALDH^-^CD24^-^CD44^+^ and bulk (non-ALDH^+^CD24^-^CD44^+^) tumor cell populations. Using gene expression profiling of ALDH^+^ and CD24^-^CD44^+^ BCSCs (comparing to bulk tumor cells) isolated across different subtypes of human breast cancer tissues together with multi-marker immunofluorescence including CD24, CD44 and ALDH1, we have recently shown that the CD24^-^CD44^+^ and ALDH^+^ cell populations identify anatomically distinct breast CSCs with distinct EMT (epithelial-to-mesenchymal transition) and MET (mesenchymal-to-epithelial transition) gene-expression profiles respectively [8]. The EMT-like CD24^-^CD44^+^ breast CSCs are primarily quiescent and localized at the tumor invasive front, while the MET-like ALDH^+^ breast CSCs are proliferative cells located mainly in the central part of tumors. Importantly, the epithelial and mesenchymal states of breast CSCs are not static; instead they display a cellular plasticity allowing them to transit between EMT and MET states [8]. This reversible, metastable epithelial-mesenchymal plasticity of breast CSCs builds upon the current model of cancer metastasis postulating that EMT drives tumor cell dissemination while subsequent MET drives metastatic colonization [27].

### Targeting two states of cancer stem cells

#### Cancer stem cell and drug resistance

A body of literature has shown the EMT type of CSC plays a critical role in drug resistance and cancer metastasis, which could partially explain why it has traditionally been difficult to cure cancer using cytotoxic chemotherapy. For instance, recent studies have shown that EMT breast CSCs were particularly resistant to treatments of either chemotherapy or radiation therapy in cell lines and patient-derived mouse xenografts [6],[25],[28],[29]. The molecular mechanisms by which the CSCs possess increased drug resistance are still not completely clear. The proteins of the ABC transporter family are believed to function as drug efflux pumps and increased levels of these proteins in CSCs could therefore be contributing to the protection of CSCs from cytotoxic chemotherapy [30]-[33]. For example, Osteosarcoma CSCs were enriched for ABCG2, a member of ABC transporter, and showed increased drug resistance and metastasis [34],[35]. Hu et al confirmed this finding by showing that high expression levels of ABCG2 in ovarian CSCs contributed significantly to Cisplatin resistance [36]. Another member of ABC transporters, MDR1/ABCCB1, has been reported to be associated with a drug-resistant profile and high clonogenic activity of the stem cell population in small-cell lung carcinoma [37]. A recent study showed that transcription factor Oct1 directly regulates ABC transporter genes *Abcg2*, *Abcb1* and *Abcb4* in CSCs [38]. In this study, the authors found an elevated protein level of Oct1, but not mRNA, highly correlated with the frequency of CD24^-^CD44^+^ EMT breast CSCs or ALDH^+^ MET breast CSCs.

There is mounting evidence suggesting that CSCs employ alternative DNA repair pathways to maintain genomic stability and prevent damage from radiotherapy. A previous study reported CD24^-^CD44^+^ EMT breast CSCs are a relatively radioresistant subpopulation and increase in numbers after short courses of fractionated irradiation [5]. Another group reported that CD24^-^CD44^+^ EMT breast CSCs isolated from either MCF7 or MDA-MB231 breast cancer cell lines, or primary culture of patient tumors, displayed increased capacity for sphere formation and resistant to radiation compared to the non- CD24^-^CD44^+^ subpopulation through the enhanced activation of DNA damage response [39]. CD133^+^ brain tumor stem cells in glioblastoma have reduced sensitivity to radiation-induced apoptosis [15],[40] and similar findings were made by Bao et al that CD133^+^ tumor stem cells isolated from both human glioma xenografts and primary patient glioblastoma specimens preferentially activate the DNA damage response after radiation treatment and repair radiation-induced DNA damage more effectively than CD133^-^ bulk tumor cells [41]. These results also suggest that the DNA repair machinery may be an effective target to eliminate CSCs, leading to increased patient survival.

Aldehyde dehydrogenase1(ALDH1) is a detoxification enzyme involved in the oxidization of intracellular aldehydes to carboxylic acids [42]. Elevated expression level of ALDH1 in MET type of breast CSCs is thought to be responsible for drug resistance to cytotoxic agents, such as cyclophosphamide [43]. Studies have shown that ALDH^+^ MET breast CSCs exhibit similarly enhanced metastatic and drug resistant features with EMT type of breast CSCs [14]. In this study, the ALDH^+^ CSC population was found to be a prognostic indicator for high tumor grade, negative ER/PR status, ERBB2 overexpression, and expression of basal-like cytokeratins. Beside those findings, ALDH1 activity in breast CSCs was considered as a predictor of poor clinical outcome [14]. Consistent with those observations, a clinical study examining ALDH1 expression level in primary breast tissue treated with Paclitaxel and Epirubicin revealed that ALDH1 activity was strongly associated with a low pathologically complete response and high resistance to chemotherapy [26]. Most recently, it has been shown that ALDH enzymes play a directed role in chemotherapy resistance [44]. Therefore, ALDH1 not only serves as a functional marker of CSCs, but also plays important role in resistance to chemotherapy.

### Targeting signaling pathways of cancer stem cells

#### The Wnt pathway

The Wnt signaling pathway is a key developmental pathway involved in a variety of biological processes including cell proliferation, survival and differentiation [45]. The well characterized canonical Wnt/β-catenin signaling pathway is initiated with ligand-receptor binding resulting in transcriptional activation of a subset of response genes, which have been suggested to play a critical role in tumor initiation in many tissues. The Wnt/β-catenin signaling pathway is often aberrantly activated in CSCs, which are responsible for generation of metastasis and decreased survival of patients [46]. Therefore, targeting the Wnt/β-catenin signaling pathway may potentially reduce the number of, or even eradicate, CSCs. To this end, a number of small-molecule inhibitors of Wnt signaling are being studied including existing drugs such as nonsteroidal anti-inflammatory drugs (NSAID), new molecular-targeted agents, including many that are currently in the discovery, preclinical, or clinical testing stages [47]. Among them, CBP/β-catenin antagonist ICG-001ICG-001, was reported to be capable of eliminating imatinib-resistant leukemic stem cells both *in vitro* and mouse xenografts. In Wilm’s tumor, the stem cell properties of sphere formation and clonogenicity can be largely abrogated after the application of anti-FZD7, an antibody for Wnt receptor Frizzled 7 [48]. Moreover, application of Dkk1 has been shown to decrease mammosphere formation in primary breast cancer cells and MCF-7 cells and drive CD24^-^CD44^+^ EMT breast CSCs into differentiation at a high concentration by preventing the formation of FZD-Wnt-LRP complex [49]. Recently, it has been demonstrated that salinomycin is able to dramatically reduce the number of CSCs in triple negative breast cancer [50] by promoting the degradation of Wnt co-receptor LRP6 [51]. Thus, targeting the components in Wnt signaling pathway is a promising approach to eliminate CSCs, especially the CSCs with EMT characteristics.

### The Hedgehog pathway

Recent studies have suggested that the Hedgehog (Hh) pathway is involved in the maintenance of CSCs in a number of tumors, including pancreatic cancer [52], gastric cancer [53], colorectal cancer [54], and glioma [55]. An Hh pathway inhibitor GDC-0449(Vismodegib) has been developed to inhibit the signaling component Smoothened (SMO) and has shown promise in clinical trials of advanced basal cell carcinoma and advanced metastatic medulloblastoma [56],[57]. However, mutations in SMO in patients conferred a drug resistance to this inhibitor [58]. An active compound in green tea, (-)-epigallocatechin-3-gallate (EGCG), has been proposed to inhibit self-renewal capacity of pancreatic CSCs through blocking Hh pathway [52]. In this study, the expression levels of sonic Hh signaling components Patched (Ptch), SMO, Gli1 and Gli2 were downregulated, and the transcriptional activities of Gli1 and Gli2 were also inhibited after application of EGCG. However, the direct target(s) of EGCG in CSC inhibition are still unclear. A small-molecule Hh pathway inhibitor, IPI-269609, has been tested both *in vitro* and *in vivo* model systems of pancreatic cancer and the results showed that this SMO inhibitor is effective in reducing ALDH^+^ MET CSCs and preventing metastasis [59]. Moreover, treatment with the Hh pathway inhibitor cyclopamine combined with gemcitabine and rapamycin showed significant inhibitory effects on CD133^+^ CSCs of human pancreatic cancer [60], suggesting that combined targeted treatment may have therapeutic efficacy. Bmi-1 is an oncogene and may cooperate with Twist1 to regulate EMT state of CSCs and metastasis. Bmi-1 has been shown to play an important role in the regulation of stem cell self-renewal in breast cancer, where the activity of Bmi-1 is increased by Hh pathway activation [61]. A recent study demonstrated that a small molecular inhibitor, PTC-209, is capable of inhibiting Bmi-1, further blocking self-renewal of colon cancer initiating cells (CICs) [62],[63], which provides a therapeutic rationale for exploring the efficacy of PTC-209 in targeting CSCs.

### The Notch pathway

The role for Notch signaling in cancer was demonstrated by the identification of activating mutations as well as amplification of Notch pathway components in a number of tumors [64],[65]. Among them, activating mutations of Notch-1 have been identified in more than 50% of human T-cell acute lymphoblastic leukemia patients (T-ALL) [66]. Overexpression of receptor Notch-1, or its ligand Jagged-1, has been shown to predict poor survival of breast cancer patients [67],[68], suggesting a significant role of the Notch pathway in this malignant disease. Moreover, Notch activated HER2 overexpression is essential for self-renewal of breast CSCs in patients and is associated with poor prognosis [69]. Blocking the activity of Notch intracellular domain (NICD) is currently one of the practical approaches to inhibiting Notch signaling to eliminate CSCs, and this therapeutic inhibition of Notch signaling can be achieved using γ-secretase inhibitors (GSIs) to block the release of NICD. GSIs were shown to reduce glioblastoma CSCs [70] and breast CSCs [71] through inhibition of Notch pathway. In another study, GSI MRK-003 has been shown to inhibit CSCs in a mouse model of breast cancer [72]. A blocking antibody or siRNA knockdown of Notch-4 has been shown to reduce the stem cell population, inhibiting tumor formation from EpCAM^+^CD24^-^CD44^+^ CSCs and that this effect was greater than Notch-1 inhibition [71],[73], this suggests that Notch4-targeted therapies may be more effective than Notch1-targeted therapies in targeting breast CSCs.

### PI3K/AKT/mTOR pathway

The PI3K/AKT/mTOR pathway has been shown to play an important role in the regulation of CSCs. PTEN acts as a negative regulator of PI3K/Akt/mTOR signaling through dephosphorylating PIP3, a product of PI3K, resulting in pathway inhibition [74]-[77]. Loss of PTEN activity leads to constitutive PI3K/Akt pathway activation, which results in increased stem cells in breast cancer. Treatment with Akt or mTOR inhibitors has been shown to increase sensitivity of CSCs to irradiation and chemotherapy, respectively [78],[79]. Accordingly, inhibition of AKT with Perifosine reduced CSC activity as accessed by mammosphere formation. These results suggest the PTEN/PI3K/AKT/mTOR pathway could be an effectively therapeutic target in sensitizing and eliminating CSCs.

### Targeting cytokines through STAT3 and NF-κB pathways

The STAT3 and NF-κB pathways play a pivotal role in the induction and maintenance of inflammatory microenvironment of malignant diseases and tumors [80]-[82], and these pathways are activated by numerous cytokines including IL-6 and IL-8 secreted by a variety of inflammatory cells [83],[84]. In cancer patients, high levels of IL-6 and IL-8 are associated with poor outcome. IL-6 activated JAK2/STAT3 pathway was proposed to support the maintenance of CD24^-^CD44^+^ breast CSCs [85]. STAT3 and AKT activation causes the activation of NF-κB pathways in inflammatory cells leading to increased levels of Lin28 and decreased levels of miRNA let7. This in turn leads to increased IL-6 and IL-8 production in the tumor microenvironment [86],[87]. A recent study revealed that IL-6 activated Jagged1/Notch1 signaling contributes to bone metastasis of breast cancer [88], indicating that multiple pathways may be involved in the IL-6-mediated CSC regulation. Moreover, we previously reported that the IL-8 receptor CXCR1 is selectively expressed in breast CSCs, and the addition of IL-8 promotes proliferation and self-renewal of breast CSCs [89]. These results suggest blocking IL-6, IL-8 and their receptors IL-6R and CXCR1 may be an attractive therapeutic strategy.

Approximately 20-25% of human breast cancers are HER2-positive and almost 50% of patients with HER2-amplified cancer will develop trastuzumab resistance after 1-2 years of treatment [90]. Notably, PTEN deletion was identified in over 40% of those HER2-positive trastuzumab resistant breast cancer patients [91]. In a recent study, we found that PTEN inactivation in HER2-positive breast cancer cells activates an IL-6, STAT3, AKT and NF-κB involved inflammatory feedback loop, which expands EMT type of breast CSCs [87]. Moreover, it has been shown that activation of the IL6 inflammatory loop in p53^−^PTEN^−^ cancer cells is associated with reduced SOCS3 expression which serves as a negative regulator of IL6-mediated signaling, and forced expression of SOCS3 or using IL6-R blockade inhibited tumor proliferation and metastasis in mouse xenografts [92]. Through the IL6 feedback loop, trastuzumab resistance is acquired and EMT-CSCs are significantly enriched. Based on this observation, we treated HER2-positive, PTEN deleted, tumor xenografts with a combined treatment of trastuzumab and tocilizumab, a monoclonal antibody directed against IL-6R. This treatment completely overcame *de novo* and acquired trastuzumab resistance of HER2-positive PTEN-deleted breast cancer. Furthermore, reparaxin, an inhibitor of the IL-8 receptor CXCR1, is currently in a phase I clinical trial, to evaluate the strategy of adding repertaxin to paclitaxel to block the chemotherapy-induced increase in CSCs.

### Non-coding RNAs in CSC plasticity

Non-coding RNAs (ncRNAs) are RNA molecules which are transcribed from the genome, but do not encode proteins. One class of ncRNAs are microRNAs which are highly-conserved small non-coding RNAs with a length of ~22 nucleotides that serve as regulatory inhibitors for protein expression [93]. They modulate gene function through their binding to complementary regions within the 3’-UTRs of target mRNAs, leading to mRNA degradation or repression of translation. MicroRNAs have been extensively studied and regulate expression of a variety of target genes involved in different developmental processes and diseases including cancer [94]. MicroRNAs can function as oncogenes [95],[96] or tumor suppressors [97],[98]. There is mounting evidence that microRNAs are potential targets for cancer diagnosis and therapy [99]-[102]. Long non-coding RNAs(lncRNAs) are a newly identified class of non-coding RNAs longer than 200 nuceotides [93]. Although relatively few lncRNAs are well characterized compared to microRNAs, lncRNAs function in physiological and pathological processes through a variety of mechanisms such as interacting with microRNAs, mRNAs, proteins and genomic DNA [103]. LncRNAs have been implicated in many aspects of epigenetic, transcriptional and translational regulation [104]. Emerging evidence suggests that non-coding RNAs might serve as potential targets for anti-CSC therapies.

### MicroRNA regulation of cancer stem cell plasticity

The most established regulatory network of microRNAs in CSCs is the miR-200 family, which includes miR-200a, b, c, miR-141 and miR-429. Two prominent targets of the miR-200 family are ZEB1 and ZEB2 which are involved in the regulation of epithelial/mesenchymal transitions. E-cadherin is one of the key epithelial genes, and downregulation of E-cadherin is generally associated with EMT. miR200-family members act to maintain E-cadherin expression through directly suppressing the negative regulators ZEB1 and ZEB2 in EMT CSCs. Forced expression of miR-200 family members was shown to prevent a TGF-β induced mesenchymal phenotype [105], and block tumorigenicity of CD24^-^CD44^+^ CSCs [106]. Consistent with this result, miR-200 family members were found to be strongly suppressed in CD24^-^CD44^+^ breast CSCs which is associated with an EMT state [106],[107]. In addition, inhibition of miR-200 family members was also shown to promote CSC formation and maintenance [108]. miR-200c inhibited the polycomb gene Bmi-1 in breast CSCs, leading to reduced self-renewal of CSCs [109]. In contrast, loss of miR-200b promotes the expression of the E-cadherin suppressor Suz12, which resulted in increased numbers of EMT CSCs [108]. In breast cancer patients, the expression of miR-200 family members inversely correlated with the proportion of CD24^-^CD44^+^ EMT breast CSCs. Furthermore, forced expression of miR-200 genes in those EMT CSCs resulted in a conversion of these CSCs to an epithelial MET state [110].

The microRNA let-7 family regulates stem cell self-renewal and differentiation and acts as a tumor suppressor through targeting oncogenic RAS and HMGA2, which are involved in the EMT state of CSCs. Overexpression of let-7 leads to suppression of mammosphere formation, proliferation, and reduced proportion of breast CSCs, suggesting let-7 inhibits cancer growth through regulation of the EMT state of CSCs [111]. Similar to the let-7 family, overexpression of miR-30 in breast CSCs resulted in reduced self-renewal and anoikis resistance and increased apoptosis via decreased Ubc9 levels and silencing of ITGB3. When both let-7 and miR-30 were expressed in breast cancer, a much more significant inhibition of self-renewal of breast CSCs was observed compared to expression of either miRNA alone [112], indicating multiple miRNAs could be used to eliminate CSCs in anti-cancer therapy. miR-34c has been identified as a tumor suppressor because it has inhibitory activity in regulating self-renewal and EMT of breast CSCs through targeting Notch4. The miR-181 family which interacts with the TGF-β pathway functions in the regulation of EMT breast CSCs [113]. Finally, miR-21 functions as an oncogenic miRNA and promotes the EMT-like breast CSCs through AKT/ERK1/2 inactivation by targeting PTEN [114].

Recently miR-93 [115], miR-100 [116], and miR-221 [114] were shown to be important regulators of the transition between EMT and MET CSC states. Low mir-93 expression is associated with increased tumor-initiating capacity, while overexpression diminishes the presence of CSCs. Forced expression of miR-93 in claudin^low^ SUM159 and basal HCC1954 cell lines, as well as NOD/SCID mouse xenografts, dramatically reduced the proportion of ALDH^+^ MET-like breast CSCs and overexpression of miR-100 in breast cancer cell lines and tumor xenografts also modulates the MET and EMT breast CSC states [115]-[116]. A number of targets of miR-93, including JAK1, SOX4, STAT3, AKT, EZH1, and HMGA2 are known regulators of stem cell-renewal. Expression of miR-93 was also found to suppress the TGF-β signaling pathway through targeting TGF-βR2 and SMAD5 to promote the conversion of EMT to MET, resulting in an increased proportion of ALDH^+^ MET breast CSCs. In contrast, forced expression of miR-100 and miR-221in MCF10A, a non-tumorigenic breast cell line, as well as a variety of tumorigenic breast cancer cell lines resulted in the induction of CD24^-^CD44^+^ EMT stem cells and decreased proportion of ALDH^+^ MET stem cells [115].

### LncRNA regulation of cancer stem cell plasticity

Hotair (Hox transcript antisense intergenic RNA) is one of the first lncRNAs whose function has been elucidated. It plays a pivotal role in the polycomb repressive complex 2 (PRC2), which directs epigenetic regulation of target genes through histone H3 lysine 27 trimethylation (H3K27me3) [117],[118]. High expression level of Hotair contributes to metastasis and poor survival in breast cancer patients [117]. Hotair is also highly expressed in CD133^+^CD44^+^ colon cancer cells which have been characterized as EMT CSCs [120]. Overexpression of Hotair resulted in increased expression of EMT inducing genes such as ZEB1, Snai1, Twist, β-catenin, vimentin and fibronectin in breast CSCs [118],[120]. Those results suggest Hotair regulates EMT CSCs and promotes cancer metastasis through global reprogramming of chromatin states.

Xist, one of the lncRNAs involved in X chromosome inactivation(XCI), was demonstrated to be a tumor suppressor and a stem cell regulator [121]. Using mathematical modeling, SUZ12 and EZH2, regulators of CSCs, were predicted to show high binding affinity to Xist RNA [122], which is further evidence that Xist may function in regulation of CSCs [123]. Salvado et al. validated that low Xist expression in patient derived xenografts is associated with drug response and a significant decrease of the ALDH^+^ breast CSC population after treatment with a histone deacetylase inhibitor (HDACi) abexinostat. In contrast, high expression of Xist in patient derived xenografts increased the proportion of breast CSCs. This result indicates that Xist’s regulation of the proportion of CSCs may be dependent on CSC states. Our laboratory identified BRCA1 as a regulator of ALDH^+^ CSCs [124], while a previous study showed that Xist RNA concentration in XCI was increased by BRCA1 in breast cancer cells [125], suggesting Xist regulation of breast CSCs is in a BRCA1 dependent manner.

Translational regulatory RNA (treRNA) was found to function in the nucleus as a *cis* element to upregulate the expression of Snail while suppress cytoplasmic E-cadherin expression promoting EMT in cancer cells [126],[127]. Overexpression of lncRNA treRNA in MCF7 breast cancer cells suppressed E-cadherin and other epithelial proteins and increased the protein levels of mesenchymal proteins, resulting in increased cell migration and invasion [127]. These results suggest treRNA may play a role in the regulation of EMT CSCs and may thus be an appropriate therapeutic target. The metastasis–associated lung adenocarcinoma transcript 1(MALAT 1) was found to be associated with highly metastatic tumors and correlated with poor patient outcome [128]. In bladder cancer, downregulation of MALAT-1 led to the inhibition of the EMT associated genes ZEB1, ZEB2, and Slug, and the activation of E-cadherin. In addition, increased expression of MALAT-1 was also found to activate the Wnt pathway to promote EMT and human bladder cancer cell metastasis [129].

LncRNA H19, together with its partner miR-675, a miRNA embedded in its first exon, were proposed to regulate EMT through multiple signaling pathways, one of them being the PI3K–AKT pathway [130]. Over-expression of H19 in lung cancer lines abolished the expression of E-cadherin by activation of Slug in a miR-675 dependent manner, suggesting a biological function of H19 in the regulation of EMT. A recent study showed that H19 can serve as a molecular sponge for the let-7 microRNA family in muscle tissue, suggesting an alternative function of lncRNAs in the transition between EMT to MET of CSCs through blocking microRNAs [131]. Linc-ROR was first characterized in induced pluripotent stem cells (iPSCs) [132] and recently it was proposed to act as a sponge for mir-145 serving to de-repress a number of mir-145 targets, including OCT4, SOX2, and Nanog [133]. More recently, linc-ROR has been demonstrated to play an important role in the regulation of breast cancer metastasis and EMT CSCs. Overexpression of linc-ROR in mammary epithelial cells resulted in an increase in the CD24^-^CD44^+^ stem cell population with strong upregulation of the mesenchymal markers such as Vimentin, α-SMA, N-cadherin and Fibronetin, while the epithelial markers E-cadherin and Occludin were dramatically suppressed [134]. In this study, linc-ROR was suggested to have a role as a sponge for mir-205 thereby preventing the degradation of its targets such as ZEB1 and ZEB2 in breast cancer to regulate CD24^-^CD44^+^ EMT stem cells. Recently, lncRNA-activated by TGF-β (lncRNA-ATB) was demonstrated to competitively bind the miR-200 family and sequester them from their targets, ZEB1 and ZEB2, thereby inducing EMT and invasion in hepatocellular carcinoma. On the other hand, lncRNA-ATB also interacts with, and increases the stability of IL-11 mRNA, which results in the activation of the IL-11-STAT3 signaling pathway and enhanced colonization in hepatocellular carcinoma [135]. These findings suggest that lncRNA-ATB might act as a key regulator of TGF-β signaling by targeting both EMT and MET states of CSCs. Future studies will further elucidate the role of lncRNAs in CSC signaling and plasticity.

## Conclusions

**Figure 1:**
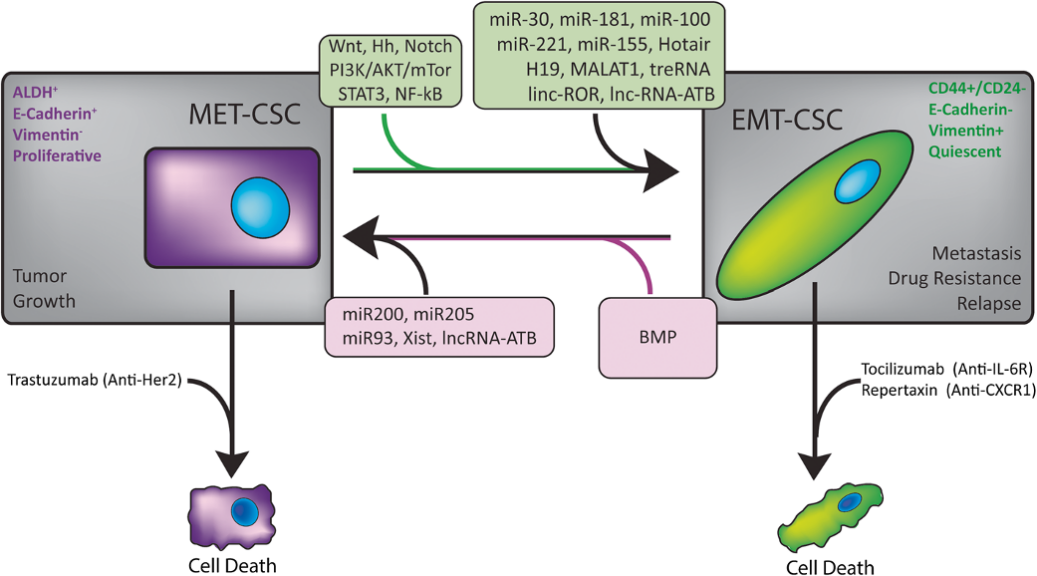
**Potential therapeutic targets of CSC plasticity.** MET and EMT CSCs can readily transition back and forth between the two cell states. Potential targets for eliminating these populations include targeting each specific population with particular drugs such as Trastuzumab(MET CSCs) or Tocilizumab (EMT CSCs) or developing new drugs to target the pathways and ncRNAs involved in the transition between the populations.

The current model of CSC plasticity between EMT and MET states suggests that CSCs may not constitute fixed populations but rather a dynamic, plastic and phenotypic state that can be acquired as a function of dynamic tumor microenvironment changes such as growth factor and inflammatory signaling, stromal cell interactions, and tumor hypoxia/metabolic reprograming. This epithelial-mesenchymal plasticity of CSCs adds another layer of complexity in terms of therapeutic strategies to target these lethal seeds of tumors. However, no matter how plastic and dynamic, CSCs are still regulated by and are depended on specific stem cell signaling pathways, and thus may be amenable to CSC specific therapies. The plasticity of CSCs is regulated by a plethora of factors, such as many signaling pathways, transcription factors, miRNAs and lncRNAs. Thus, targeting both EMT and MET states of CSCs may prove to be the most effective therapeutic strategy for anti-cancer treatment (Figure 1). Emerging evidence has shown that lncRNAs may play roles in the regulation of the EMT and MET states of CSCs. However, lncRNAs have been characterized to be involved in diverse physiological and pathological processes through multiple targets and distinct molecular mechanisms. Therefore, the therapeutic potential of lncRNAs in control of EMT and MET states of CSCs needs to be validated in future studies.

## Author’s contributions

YZ and ML wrote the manuscript. MB created Figure 1. All the authors read, approved and edited the final manuscript.

## Authors’ original submitted files for images

Below are the links to the authors’ original submitted files for images. Authors’ original file for figure 1

## Abbreviations

TIC: Tumor-initiating cells
CSC: Cancer stem cell
EMT: Epithelial-to-mesenchymal transition
MET: Mesenchymal-to-epithelial transition
ALDH: Aldehyde dehydrogenase
ABC: ATP-binding cassette
MDR: Multi-drug resistance
ncRNA: Non-coding RNA
lncRNA: Long non-coding RNA
