# Supplementary figures and images for: Biological and clinical significance of cancer stem cell plasticity
Source: Clin Transl Med. 2014 Oct 7;3:32. doi: 10.1186/s40169-014-0032-3 (PMC4883980; doi:10.1186/s40169-014-0032-3)

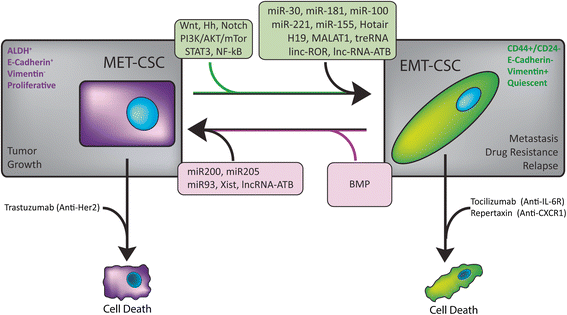

Supplement: Supplementary file 1 — Authors’ original file for figure 1 [file 40169_2014_32_MOESM1_ESM.gif]
